# *LMNA*-mutated Rabbits: A Model of Premature Aging Syndrome with Muscular Dystrophy and Dilated Cardiomyopathy

**DOI:** 10.14336/AD.2018.0209

**Published:** 2019-02-01

**Authors:** Tingting Sui, Di Liu, Tingjun Liu, Jichao Deng, Mao Chen, Yuanyuan Xu, Yuning Song, Hongsheng Ouyang, Liangxue Lai, Zhanjun Li

**Affiliations:** ^1^Jilin Provincial Key Laboratory of Animal Embryo Engineering, Jilin University, Changchun 130062, China; ^2^Key Laboratory of Regenerative Biology, and Guangdong Provincial Key Laboratory of Stem Cells and Regenerative Medicine, South China Institute for Stem Cell Biology and Regenerative Medicine, Guangzhou Institutes of Biomedicine and Health, Chinese Academy of Sciences, Guangzhou, Guangdong 510530, China

**Keywords:** CRISPR/Cas9, *LMNA*, premature aging syndrome, rabbit

## Abstract

Premature aging syndromes are rare genetic disorders mimicking clinical and molecular features of aging. Products of the *LMNA* gene, primarily lamin A and C, are major components of the nuclear lamina. A recently identified group of premature aging syndromes was related to mutations of the *LMNA* gene. Although *LMNA* disorders have been identified in premature aging syndromes, affect specifically the skeletal muscles, cardiac muscles, and lipodystrophy, understanding the pathogenic mechanisms still need to be elucidated. Here, to establish a rabbit knockout (KO) model of premature aging syndromes, we performed precise *LMNA* targeting in rabbits via co-injection of Cas9/sgRNA mRNA into zygotes. The *LMNA*-KO rabbits exhibited reduced locomotion activity with abnormal stiff walking posture and a shortened stature, all of them died within 22 days. In addition, cardiomyopathy, muscular dystrophy, bone and joint abnormalities, as well as lipodystrophy were observed in *LMNA*-KO rabbits. In conclusion, the novel rabbit *LMNA*-KO model, displayed typical features of histopathological defects that are observed in premature aging syndromes, and may be utilized as a valuable resource for understanding the pathophysiological mechanisms of premature aging syndromes and elucidating mysteries of the normal process of aging in humans.

Nuclear lamins are type V intermediate filament proteins that are implicated in a variety of cellular processes, including DNA replication, gene transcription and chromatin organization [1-4]. Three major lamins (A, B1, and C), and several minor lamins (B2 and A△10), found in adult mammalian somatic cells. These various forms are grouped into two classes, A-type (A, A△10 and C) and B-type (B1 and B2) [5, 6]. B-type lamins are found in both embryonic and differentiated cells, whereas A-type lamins are only expressed in later stages of development and in differentiated cells [7].

The nuclear lamin A (*LMNA*) gene encodes A-type lamins, consists of 12 exons on chromosome 1, and encodes two components of the nuclear envelope (lamins A and C). The two proteins form heterodimers through their rod domains, and produce the filamentous structures found in the nuclear lamina [6]. Previous studies have reported that mutations in the *LMNA* gene are associated with a wide range of human genetic disorders, including dilated cardiomyopathy with conduction-system disease (DCM-CD) [8], limb girdle muscular dystrophy with atrioventricular conduction disturbance (LGMD1B) [9], Dunnigan-type of familial partial lipodystrophy [10], autosomal recessive Charcot-Marie-Tooth disease type 2 [11], mandibuloacral dysplasia[12], Hutchinson-Gilford progeria syndrome (HGPS) [13, 14], atypical Werner’s syndrome [15], and restrictive dermopathy [16]. Some phenotypic overlaps between the various types of laminopathies have also been observed [17, 18].

To date, several mouse models were established with genetically engineered modified *LMNA*. Lamin A/C null mice (Lmna^-/-^) presented with numerous defects, including growth retardation, muscular dystrophy, and weakening of the cardiac muscle [19]. Lmna^L530P/L530P^ mice showed clinical defects that were consistent with human HGPS, including a reduction in growth rate, pathologies of the bone, muscle and death by 4 weeks of age [20]. However, these mouse models failed to recapitulate several features of the premature aging syndrome from the gene mutation to the clinic. For example, Lmna^H222P/H222P^ mice did not exhibit biochemical features of lipodystrophy [21]. Furthermore, it was not distinct for the pathophysiology and therapeutic strategies of the devastating premature aging syndrome. Therefore, there is a need to develop premature aging syndrome models in species other than the mouse, which recapitulate human premature aging syndrome with reasonable costs for ordinary laboratory.

To our knowledge, rabbits share more similarities with humans regarding physiology, anatomy, and genetics when compared to mice [22]. In addition, rabbits have a bigger body size and a longer lifespan than mice and are also easy to handle. These advantages have contributed to rabbits becoming an appropriate animal model for cardiovascular and metabolic diseases research.

In the present study, rabbits were chosen to establish a novel progeria model by cytoplasm microinjection of Cas9 mRNA and gRNA. These *LMNA* knockout (KO) rabbits displayed typical features of premature aging syndromes, including cardiac and skeletal muscle degeneration with fibrosis, impaired mobility, lipodystrophy, and bone abnormalities. In conclusion, this novel rabbit *LMNA* deficiency model may be a valuable resource for premature aging and preclinical studies.

## MATERIALS AND METHODS

### Animals and ethics statement

New Zealand rabbits used in this study were gained from the Laboratory Animal Centre of Jilin University (Jilin, China). All experiments involving rabbits were approved by the Animal Care Center and Use Committee of Jilin University (Jilin, China). All methods were performed in accordance with the approved guidelines.

### sgRNA design and vector construction

sgRNAs were designed and assembled as previously described [23]. Complementary oligo sgRNAs were cloned into *BbsI* restriction sites of a Puc57-T7-sgRNA cloning vector (Addgene ID 51306). The Puc57-T7-sgRNA vector was amplified by PCR using the following T7 primers (T7-F: 5’-GAA ATT AAT ACG ACT CAC TAT A-3’ and T7-R: 5’-AAA AAA AGC ACC GAC TCG GTG CCA C-3’). Then, PCR products were *in vitro* transcribed using a MAXIscript T7 Kit (Ambion) and purified using a miRNeasy Mini Kit (Qiagen) according to the manufacturer’s instructions.

In addition, the 3x FLAG-NLS-SpCas9-NLS vector (Addgene ID 48137) was linearized with *NotI* and *in vitro* transcribed using the mMessage mMachine SP6 Kit (Ambion) and an RNeasy Mini Kit (Qiagen).

### Embryo microinjection and embryo transfer

The procedures of embryo microinjection and embryo transfer were similar as previously described [24]. In brief, female New Zealand White rabbits at 6-8 months of age were superovulated with 50 IU follicle stimulating hormone (FSH) at intervals of 12h for 6 times, mated with male New Zealand White rabbits, and injected with 100IU human chorionic gonadotropin (HCG). At 18h post HCG injection, female rabbits were euthanized, and the oviducts were flushed with 5ml DPBS-BSA to collect zygotes. Rabbit embryos at the pronuclear stage were collected. Furthermore, a mixture of Cas9 mRNA (200ng/ul) and sgRNA (50ng/ul) was microinjected into the cytoplasm of rabbit zygotes. The injected embryos were transferred into Earle’s Balanced Salt Solution (EBSS) medium at 38.5?and 5% CO_2_ for short-term culture. Finally, 30-50 injected zygotes were transferred into the oviduct of recipient rabbits.

### Mutation detection in pups by PCR

The gDNA of *LMNA*-KO and WT rabbits was extracted from a small piece of ear tissue using the TIANamp Genomic DNA Kit (Tiangen, Beijing, China) according to the manufacturer’s instructions. The mutation detection was performed by PCR using the following primers: F: 5’- GAA GGG TGG AGA GAC AGG AA-3’ and R: 5’- TCT TTC ACT CAT TGG ATG TAG C-3’. PCR products were gel purified and cloned into a pGM-T vector (Tiangen, Beijing, China). At least 10 positive plasmid clones were sequenced and analyzed using DNAman.

### T7EI cleavage assay

The T7EI assay was performed as described previously [25]. Briefly, PCR products were purified using a TIANgel Midi Purification Kit (Tiangen, Beijing, China) and were denatured and annealed in NEBuffer 2 (NEB) using a thermocycler. Hybridized PCR products were digested with T7 endonuclease I (NEB, M0302L) for 30 minutes at 37? and subjected to 2% agarose gel electrophoresis.

### Off-target analysis

Candidates for off-target sequences were selected by using the CRISPR Design tool (http://crispr.mit.edu/). PCR products were subjected to T7EI assay and Sanger sequence analysis. The primers used for this analysis are listed in Supplementary Table 1.

### RNA extraction and qRT-PCR

Total RNA was isolated from interscapular depot brown fat of WT and *LMNA*-KO rabbits using TRNzol-A+ reagent (Tiangen, Beijing, China), and treated with DNase I (Fermentas). First-strand cDNA was synthesized using the cDNA first strand synthesis kit (Tiangen, Beijing, China) and used for quantitative RT-PCR (qRT-PCR) analyses to evaluate expression of the *LMNA* gene. Primers were used for qRT-PCR are shown in Supplementary Table 2. qRT-PCR was performed using the BioEasy SYBR Green I Real Time PCR Kit (Bioer Technology, Hangzhou, China), and the 2^-ΔΔCT^ formula was used to analyze gene expression, *Gapdh* was used as a reference gene. All experiments were repeated for three times for each gene. The data are expressed as the mean ± S.E.M.

### Serum biochemistry analysis

The blood sample was obtained from the ear vein and collected in heparinized tubes. Serum samples were collected via precipitation and centrifugation. Serum levels of total cholesterol (TC) were evaluated using a total cholesterol test kit (colorimetric method), serum levels of low density lipoprotein (LDL) were measured using a low-density lipoprotein test kit (SUR method), serum levels of high-density lipoprotein were measured by a high-density lipoprotein test kit (SUR method), serum levels of triglycerides were determined using a triglycerides test kit (GPO-PAP method), serum total protein levels were measured using a total protein test kit (Biuret method), serum albumin levels were determined using an albumin test kit (Bromocresol green method), serum cystatin c levels were measured by a cystatin c levels test kit (Latex enhanced immunoturbidimetry method), and serum creatinine levels were determined by a creatinine test kit (Sarcosine oxidase method).

### X-ray absorptiometry

X-ray radiography scans of the whole body, femur, and tibia of *LMNA*-KO and WT rabbits at the same age were taken using a YEMA Radiography System with a digital camera (Varian, USA) attached to a X-ray radiographer (Rotanode, Toshiba, Japan). Images were taken at 50 KV with 3mAs exposure.

### Histological analysis

*LMNA*-KO and WT rabbits were euthanized at 18 days of age and tissue samples of cardiac muscle, aorta, skeletal muscle, tongue, diaphragm, bladder, bone, interscapular depot brown fat, liver, lung, and kidney were collected and fixed in 4% paraformaldehyde at 4?. Then, tissue was dehydrated in increasing concentrations of ethanol, 70% for 6 h, 80% for 1 h, 96% for 1 h, and 100% for 3 h, cleared in xylene, and embedded in paraffin for histological analysis. For hematoxylin and eosin (H&E) and Oil red, and Masson’s trichrome staining, 5μm sections were cut. H&E, Masson’s trichrome, and Oil red staining were performed as previously described [26, 27], and stained sections were analyzed under a light microscope (Nikon ts100)

### Echocardiography

Echocardiography assay was performed as described previously [28]. Briefly, Two-dimensional and M-mode transthoracic echocardiography were performed on WT and *LMNA*-KO rabbits using an M7 all digital color Doppler ultrasound diagnostic system (M7, Mindray, China). Rabbits were imaged in imaged right lateral recumbency by restraining their limbs from parasternal long and short axis views. A linear array probe and a center frequency of 10.0 MHz were used for imaging purposes. Cardiac dimensions, the percentage of fractional shortening (FS), left ventricular ejection fraction (EF), and heart rate were measured.

### Morphometric analysis of myofibers

Cross section of the H&E-staining of the gastrocnemius muscles from *LMNA*-KO and WT rabbits at the age of 18 days were analyzed for fiber size. A minimum of three different regions were counted per section. Fiber size and percentage of central nucleated fibers were calculated using ImageProPlus 6.0 software (Media Cybernetics, Silver Spring, MD, USA).

### Body weight, survival, and statistical analysis

Body weight of WT and *LMNA*-KO rabbits was recorded every two days at the same age, and mortality was monitored daily. Data were expressed as the mean ± SEM of at least 3 individual experiments. The data was analyzed by the Student’s t-test using Graphpad Prism software 7.0 (La Jolla, CA, USA). A probability of *p* < 0.05 was considered statistically significant.

**Figure 1.**
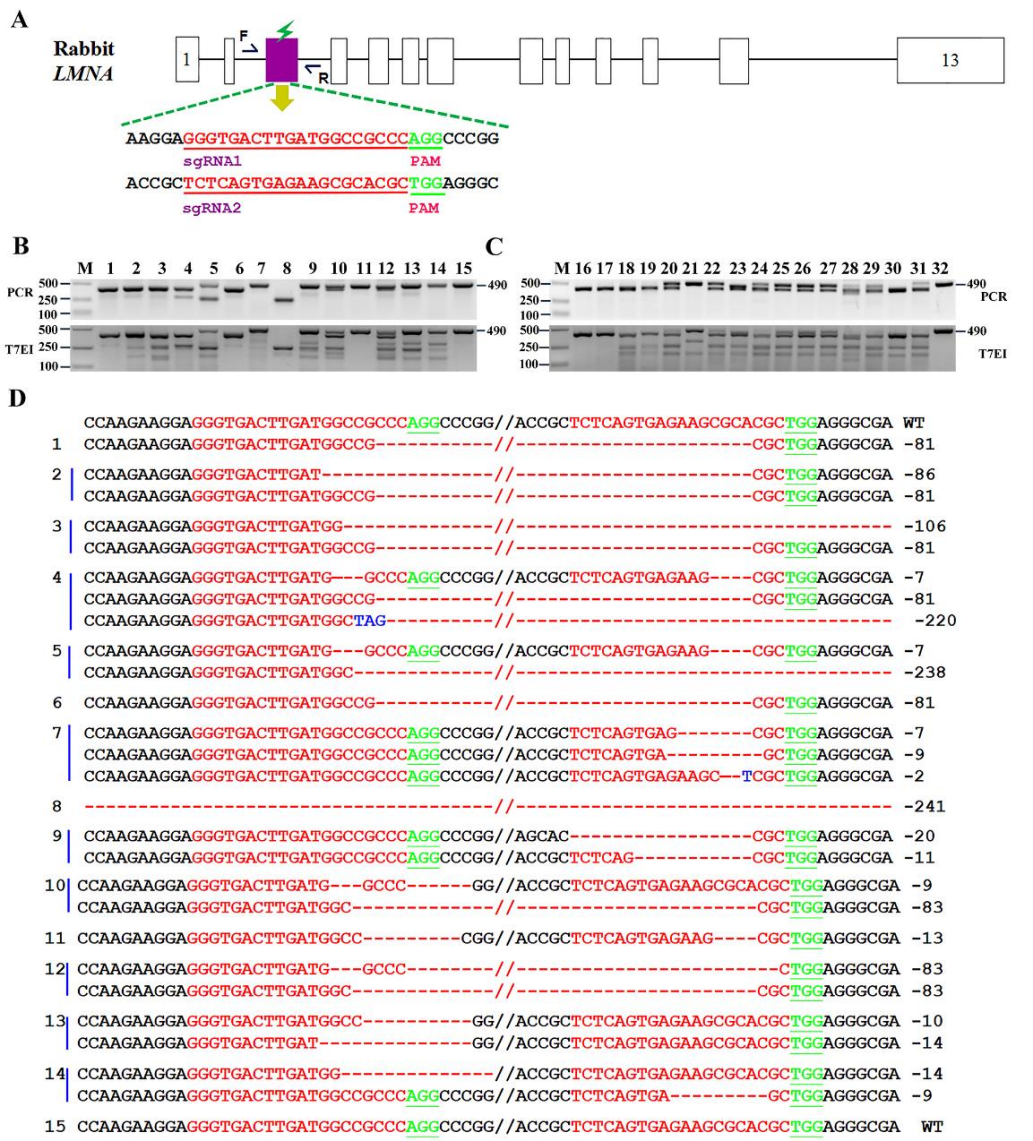
*Generation of *LMNA*-KO rabbits using CRISPR/Cas9 system*. (**A**) Schematic diagram of the two sgRNA target sites located in exon 3 of the rabbit *LMNA* locus. *LMNA* exons are indicated by pink rectangles; target sites of the two sgRNA sequences, sgRNA1 and sgRNA2, are highlighted in red; protospacer-adjacent motif (PAM) sequence is highlighted in green (**B**) Mutation detection by T7E1 cleavage assay in rabbit pups 1-15. Gel images have been cropped. M, DL2000, has used to indicate band size. (**C**) Mutation detection by T7E1 cleavage assay in rabbit pups (n=16-32 pups). Gel images have been cropped. M, DL2000, has been used to indicate band size. Black line indicates the WT allele (490 bp). (**D**) T-cloning and Sanger sequencing of modified *LMNA* alleles in 1-15 pups. WT sequence is shown at the top of the targeting sequence. PAM sites are highlighted in green; target sequences are shown in red; deletions (-); insertions are shown in blue; WT, wild-type control.

**Table 1.** Summary of embryo microinjections of Cas9/sgRNA in zygotes.

	Replications	No. zygotes	2-cell (%)	Morula (%)	Blastocyste (%)	Blastocyst with Mutantion (%)
Non-injection	3	128	(95.3±1.36)^a^	(87.5±0.77)^a^	(79.3±1.25)^a^	
Injection	3	130	(96.0±0.89)^a^	(86.7±0.99)^a^	(80.4±1.42)^a^	(95.3±0.97)^b^

a,b Different superscripts indicated significant difference (p < 0.05). Data are presented as mean ± SEM and analyzed by t-tests using GraphPad Prism software 7.0. * p < 0.05; ** p < 0.01; *** p < 0.001.

## RESULTS

### Generation of *LMNA* KO rabbits via the CRISPR/Cas9 system

To disrupt the function of *LMNA* in rabbits, we designed a pair of sgRNAs targeting exon 3 of the rabbit *LMNA* gene (Fig. 1A). Target sites are shown in Fig. 1A. Mixed Cas9 mRNA and sgRNAs were co-injected into rabbit zygotes. As shown in Table 1, 80.4% of the injected embryos developed into the blastocyst stage, and roughly 95.3% of these blastocysts carried mutations in the *LMNA* gene. No significant differences were observed in the developmental rate between non-injected embryos and CRISPR/Cas9-injected embryos (*p* > 0.05), indicating that the microinjection process itself had little to no impact on the development of the rabbit embryo.

**Figure 2.**
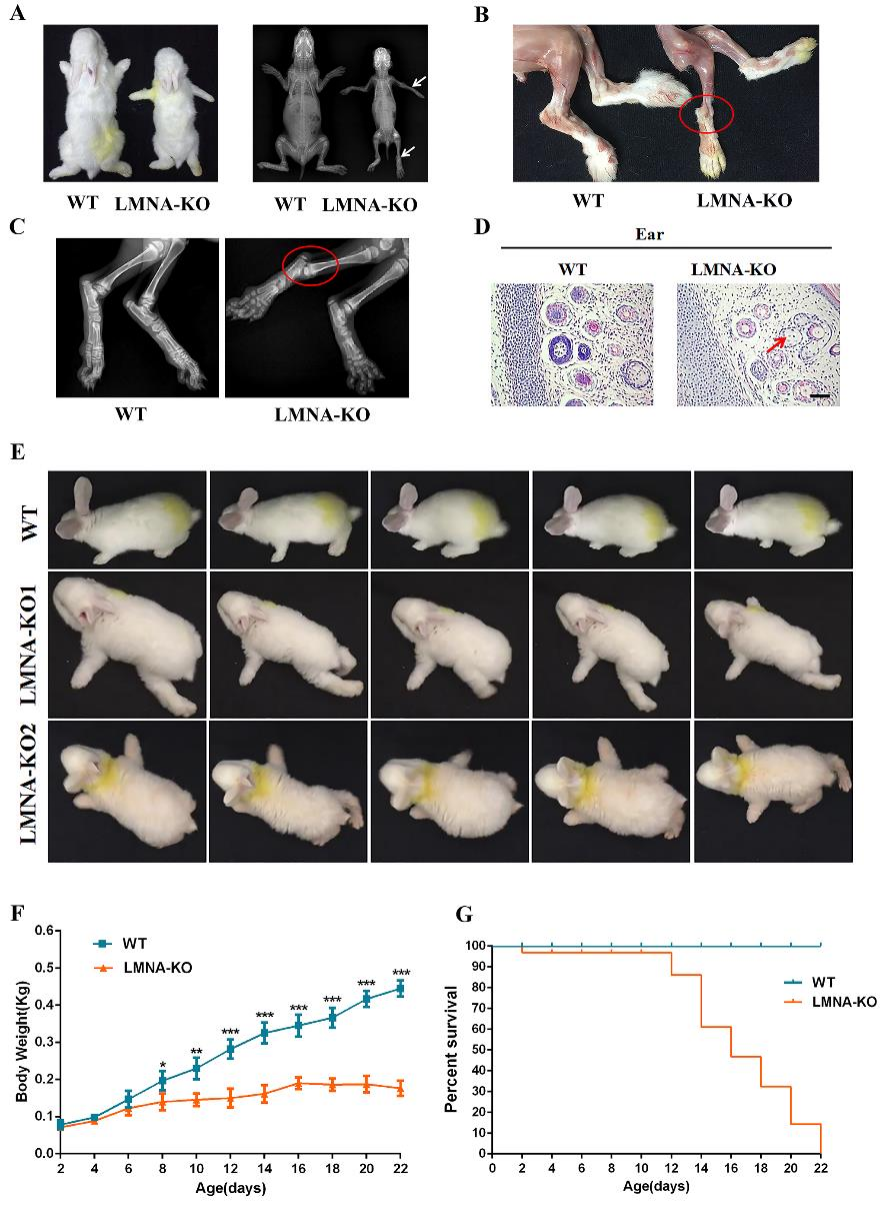
**Phenotype characterization of *LMNA*-KO rabbits**. (**A**) The gross performance of 14-day-old *LMNA*-KO rabbits by photo, and joint stiffness (white arrows) by X-ray autoradiography examination. (**B**) Hind legs of WT and *LMNA*-KO rabbits showing stiff ankle joints in *LMNA*-KO rabbits (Red oval). (**C**) X-ray absorptiometry of hind legs from WT and *LMNA*-KO rabbits showing stiff ankle joints in *LMNA*-KO rabbits (Red oval). (**D**) H&E-staining of the skin, *LMNA*-KO rabbit showed decreased eccrine in skin (**E**) Behavioral photographs cut from video of the *LMNA*-KO-1, *LMNA*-KO-2, and WT control. (**F**) Body-weight comparison of *LMNA*-KO and WT rabbits from newborn to 22 days. (**G**) Survival curves of *LMNA*-KO and WT rabbits. Scale bar, 50 μm.

**Figure 3.**
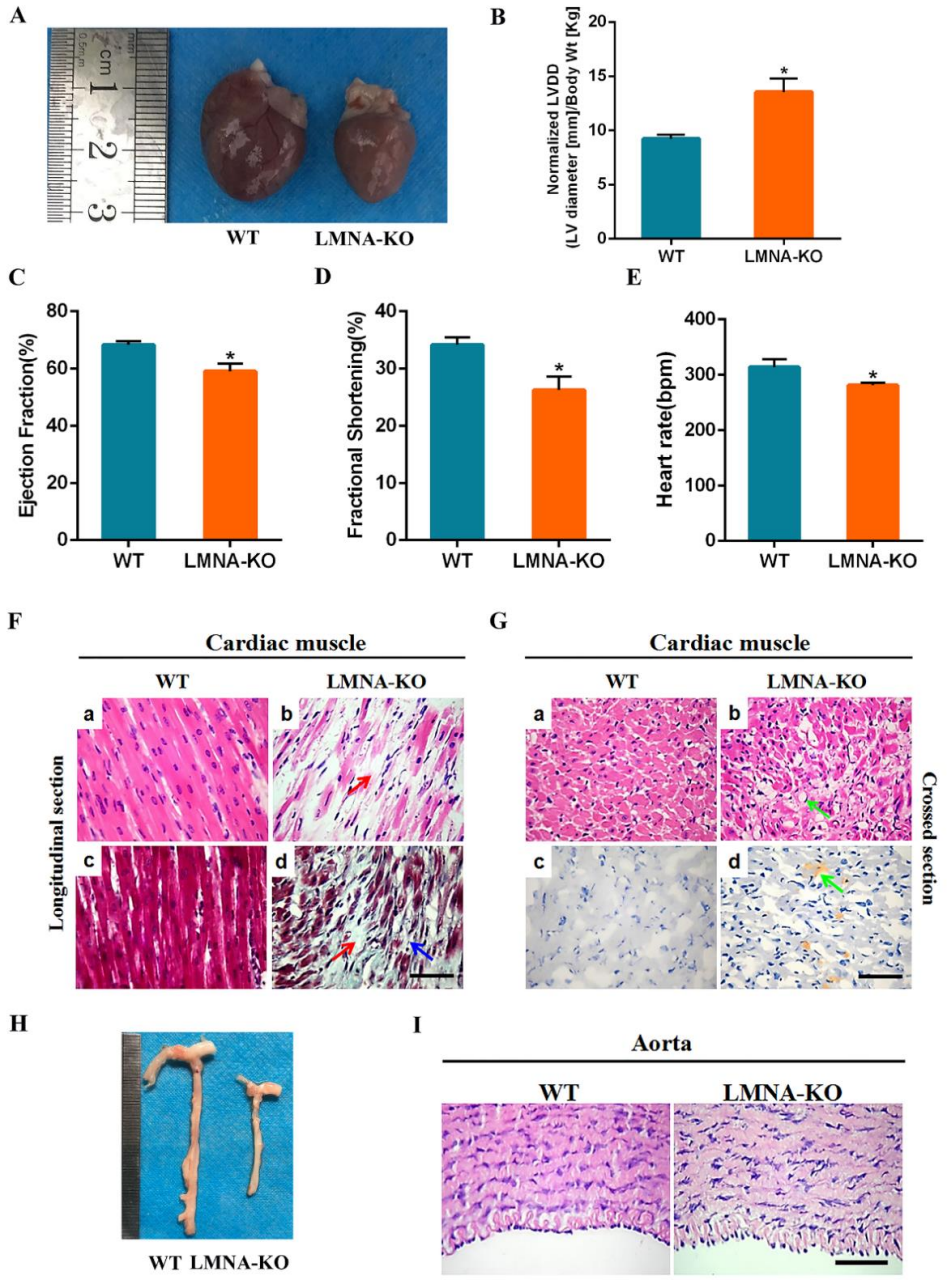
Cardiomyopathy of *LMNA*-KO rabbits. (A) The heart from 18-day-old *LMNA*-KO rabbits and WT control. (B) The increased left ventricular diastolic diameter in *LMNA*-KO rabbits. (C) The decreased left ventricular ejection fraction (EF) in *LMNA*-KO rabbits. (D) The decreased fractional shortening in *LMNA*-KO rabbits. (E) The decreased heart rate in *LMNA*-KO rabbits. Data were presented as means ± SEM of at least three rabbits per group and analyzed by Student’s *t*-tests using GraphPad Prism software 7.0. * *p* < 0.05; ** *p* < 0.01; *** *p* < 0.001. Normalized LVDD, LV diastolic diameter-to-body weight ratio. (F) H&E-staining and Masson’ trichrome-staining of cardiac muscles from WT and *LMNA*-KO rabbits, showing significant fibrosis (Red arrows) and cardiomyocytes loss (Blue arrows) in *LMNA*-KO rabbits. (G) H&E-staining and Oil red-staining of the cardiac muscles from WT and *LMNA*-KO rabbits, showing significant fat infiltration (Green arrows) in *LMNA*-KO rabbits. (H) Aorta tissues of the 18-day-old *LMNA*-KO rabbits and WT controls. (I) H&E-staining of the aorta from WT and *LMNA*-KO rabbits. Scale bar, 50 μm.

**Table 2:** Generation of *LMNA*-KO rabbits using CRISPR/Cas9 system.

Recipients	Embryos transferred	Pregnancy	Pups obtained (%transferred)	Pups with mutations (% pups)	Pups with biallelic mutations (%pups)
1	36	YES	15 (41.7%)	14 (93.3%)	13 (92.6%)
2	31	YES	7 (22.6%)	7 (100.0%)	7 (100.0%)
3	29	YES	7 (24.1%)	6 (85.7%)	6 (100.0%)
4	30	YES	3 (10.0%)	3 (100.0%)	3 (100.0%)

To establish *LMNA*-KO rabbits, a total of 126 injected zygotes (at the pronuclear stage) were transferred into the oviducts of four surrogate rabbits. All surrogates were pregnant to term and gave birth to 32 live pups (Table 2). The genomic DNA from each pup was extracted, and mutations were analyzed by Sanger sequencing. As shown in Table 2, 30 of the 32 (93.8%) newborn pups carried a *LMNA* mutation (Fig. 1B-D and Supplementary Fig. 1). These results demonstrated that the dual sgRNA-directed CRISPR/Cas9 system was efficient in generating mutations of *LMNA* gene in rabbit zygotes.

To examine off-target effects in *LMNA*-KO rabbits, the PCR products of the top ten potential off-target sites were subjected to Sanger sequencing and T7E1 cleavage assay. The results demonstrated that no off-target mutations were detected at these potential sites in *LMNA*-KO rabbits (Supplementary Fig. 2).

### Phenotype analysis of LMNA knockout rabbits

In order to characterize the phenotypes of the *LMNA*-KO rabbits, skeletal X-rays, behavioral changes, body weight and mortality rate were evaluated. Skeletal X-rays were performed and compared between *LMNA*-KO rabbits and their WT littermates. We found that *LMNA*-KO rabbits shown the joint stiffness, stiff walking posture and slight waddling gait (Fig. 2A-C and E, Supplementary movie 1, 2 and 3). Similarly, progeria patients adopt a ‘horse-riding stance’ with a wide, shuffling gait due to joint stricture, confirmed in the *LMNA*-KO rabbits [29]. In addition, when compared to WT controls, *LMNA*-KO rabbits exhibited decreased eccrine in skin (Fig. 2D), which is similar to the skin abnormalities reported in the progeria patients [30].

Additionally, no significant differences in the body weight were found between newborn *LMNA*-KO and the WT rabbits, while detectable growth retardation was observed in KO rabbits starting at 8 days of age, when compared to the WT rabbits (Fig. 2F). Besides, the *LMNA*-KO rabbits started to die after 12 days of birth, and all the KO rabbits (100.0%) died within 22 days after birth, when compared with the 0% mortality rate of the WT controls (Fig. 2G).

### Cardiomyopathy of the LMNA-KO rabbits

Dilated cardiomyopathy (DCM) is characterized by cardiac dilation and systolic dysfunction [31]. In this study, gross examination, histological and functional assessments of these rabbits were performed, to evaluate whether disruption of the *LMNA* gene in rabbits caused any cardiac-related pathological changes. The result showed that there were no significant differences between WT and *LMNA*-KO rabbits by gross examination (Fig. 3A). In addition, to determine whether disruption of the *LMNA* gene had an impact on cardiac function in KO rabbits, parameters associated with cardiac conduction were recorded using echocardiography of 18-day-old rabbits. As shown in Fig. 3B-E, *LMNA*-KO rabbits displayed significantly increased left ventricular diastolic diameters (LVDd) normalized to body weight, decreased left ventricular ejection fraction and fraction shortening compared to their WT littermates. In addition, heart rate was also decreased in *LMNA*-KO rabbits when compared to WT controls. Furthermore, *LMNA*-KO rabbits exhibited a significant loss in cardiomyocytes, interstitial fibrosis and fat infiltration of the cardiac muscle (Fig. 3F, G). However, histological analysis did not reveal any significant differences of between aortae of *LMNA*-KO and WT rabbits (Fig. 3H, I). These results confirmed a cardiomyopathy phenotype in *LMNA*-KO rabbits.

### Muscular dystrophy in LMNA-KO rabbits

**Figure 4.**
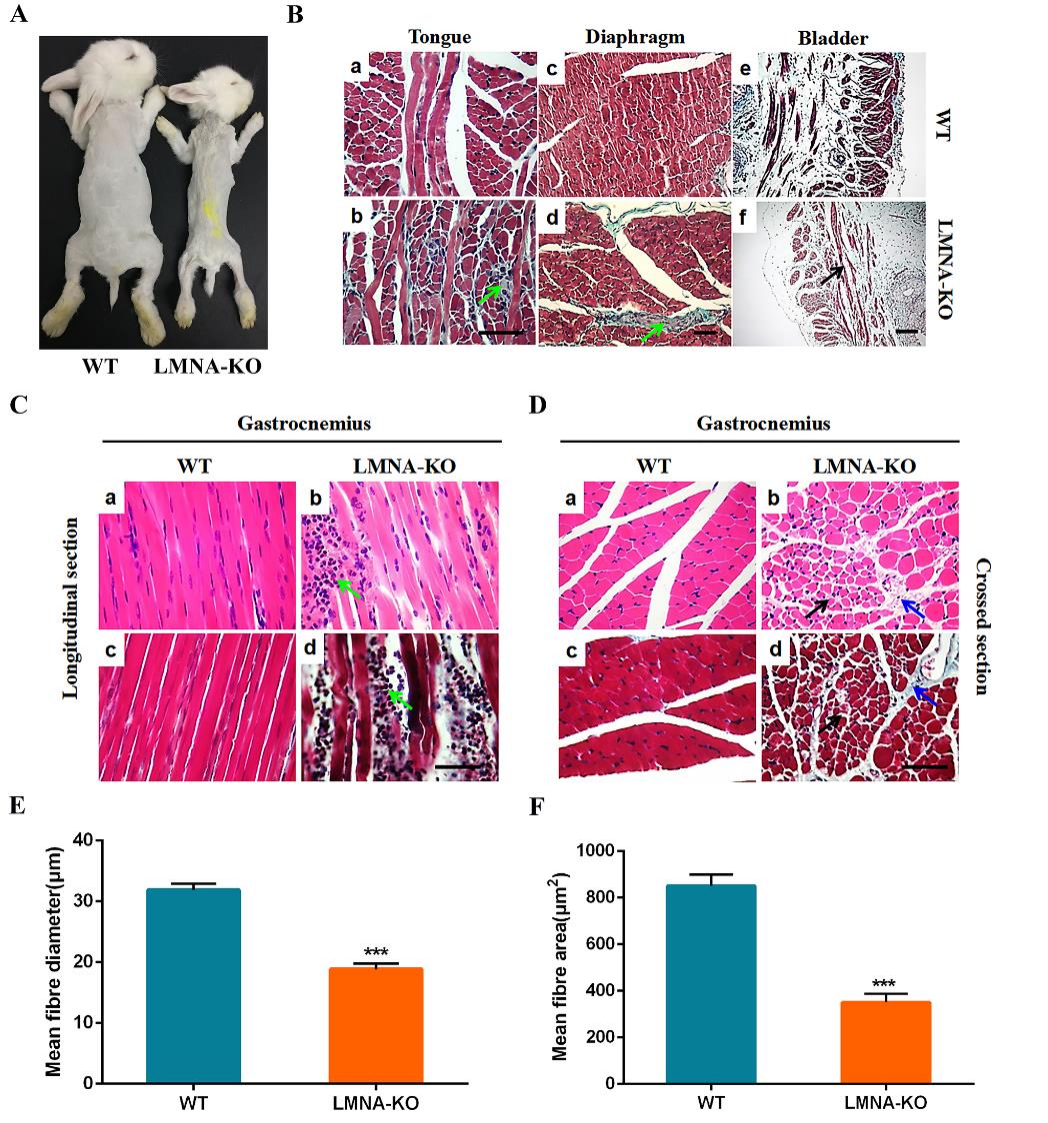
Muscular dystrophy in *LMNA*-KO rabbits. (A) Gross muscles of the 18-day-old *LMNA*-KO rabbits and WT control by photo. (B) Masson’s trichrome staining of the tongue, bladder and diaphragm muscles from WT and *LMNA*-KO rabbits, showing inflammatory cells infiltration in the tongue (Green arrows), and thinner bladder muscles of *LMNA*-KO rabbits (Black arrows). (C) Longitudinal section of the H&E-staining and Masson’s trichrome staining of the gastrocnemius muscles from WT and *LMNA*-KO rabbits, showing inflammatory cells infiltration (Green arrows) in *LMNA*-KO rabbits. (D) Cross section of the H&E-staining and Masson’s trichrome stained of the gastrocnemius muscles from WT and *LMNA*-KO rabbits, showing a wide variation in the fiber size (Black arrows), an increased number of atrophic fibers, hypertrophic fibers and lobulated fiber (Blue arrows) in *LMNA*-KO rabbits. (E) Statistical analysis of the mean fibers diameter of the muscle fibers from the gastrocnemius muscles of the 18-day-old WT and *LMNA*-KO rabbits. (F) Statistical analysis of the mean fibers area of the muscle fibers from the gastrocnemius muscles of the 18-day-old WT and *LMNA*-KO rabbits. Scale bar, 50 μm.

It has been reported that *LMNA* mutations can cause autosomal forms of Emery-Dreifuss muscular dystrophy (AD-EDMD) and limb-girdle muscular dystrophy type 1B (LGMD1B), which show clinical features of proximal dominant muscle weakness [9, 32]. As shown in Fig. 4A, *LMNA*-KO rabbits demonstrated that severe growth retardation as well as muscular dystrophy. To further investigate the pathological changes in *LMNA*-KO rabbits, we performed H&E and Masson’s trichrome staining of the muscle sections. As shown in Fig. 4B and Supplementary Fig. 3, H&E and Masson staining indicated that compared with the WT rabbits, *LMNA*-KO rabbits exhibited inflammatory cell infiltration with muscle fibrosis of the tongue muscles (Fig. 4Bb), significant fibrosis of the diaphragm muscle (Fig. 4Bd), and also thinner muscle fibres of the bladder muscle (Fig. 4Bf and Supplementary Fig. 3f). In addition, in *LMNA*-KO rabbits, typical muscular dystrophy symptoms were evidenced of the gastrocnemius as indicated by a wide variation in fiber size, including an increased number of atrophic fibers, hypertrophic fibers and lobulated fibers, as well as the regenerative muscle fibers when compared with WT littermates (Fig. 4C and D).

Additionally, morphometric analysis was calculated on gastrocnemius muscle samples of 18 days old *LMNA*-KO and WT rabbits. As shown in Fig. 4E and F, compared with the age-matched WT controls, reduced mean fibres area and diameter of muscle fibres were observed in the *LMNA*-KO rabbits. These results indicated that *LMNA*-KO rabbits demonstrated muscular dystrophy.

### Bone abnormalities of LMNA-KO rabbits

**Figure 5.**
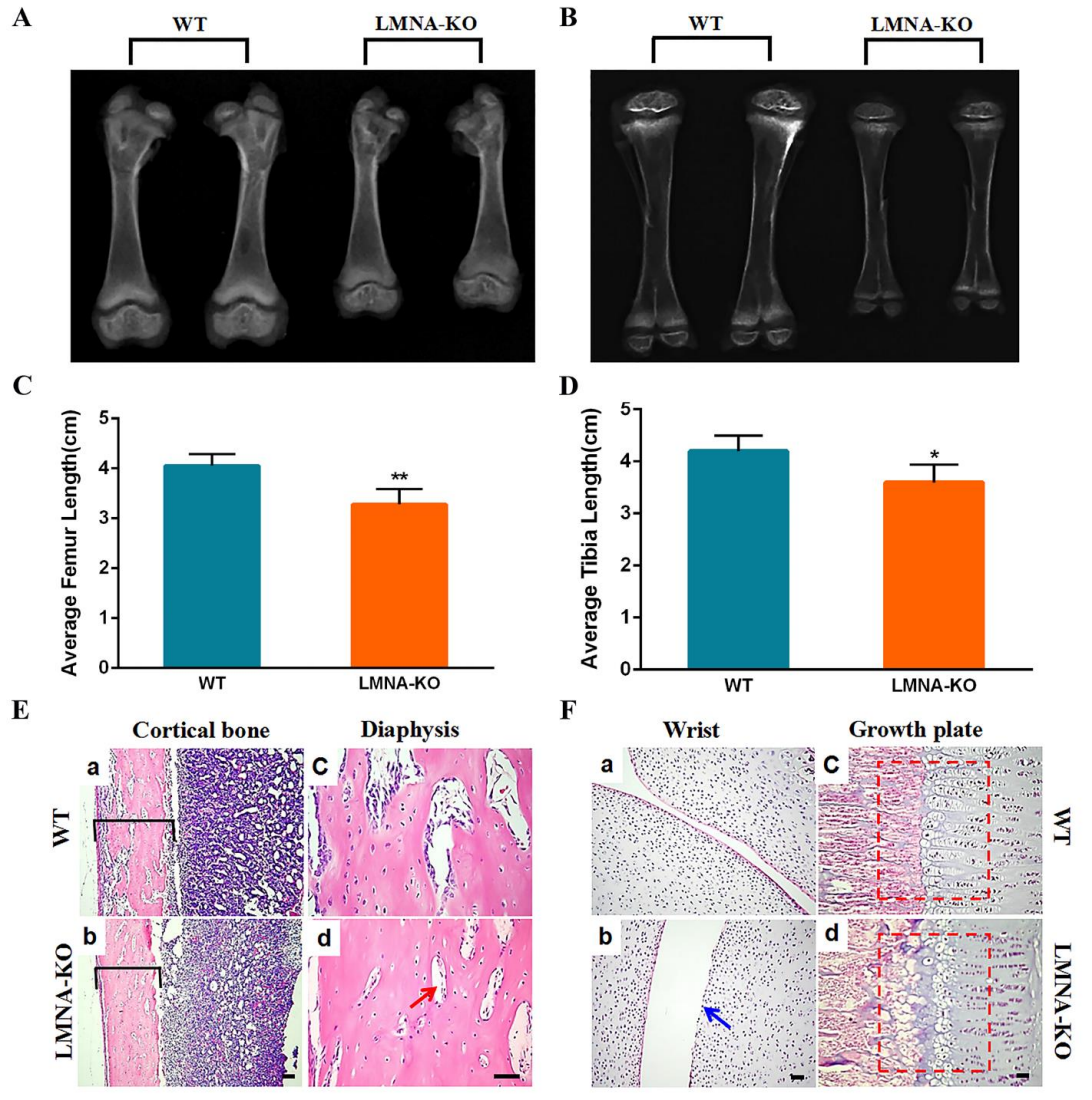
Bone abnormalities of *LMNA*-KO rabbits. X-ray absorptiometry of the femur (A) and the tibia (B) from WT and *LMNA*-KO rabbits. Statistical analysis of the average femur length (C) and tibia length (D) in WT and *LMNA*-KO rabbits. Data are presented as means ± SEM of at least three rabbits per group and analyzed by Student’s *t*-test using Graphpad Prism software 7.0. * *p* < 0.05; ** *p* < 0.01; *** *p* < 0.001. (E) H&E-staining of the cortical bone and diaphysis bone from WT and *LMNA*-KO rabbits, showing decreased cortical bone width, significantly reduced numbers of the osteoblasts and osteocytes in *LMNA*-KO rabbits. (F) H&E-staining of the wrist and growth plates from WT and *LMNA*-KO rabbits, showing rough articular surface and irregular arrangement of the growth plate with more porous areas in *LMNA*-KO rabbits. Scale bar, 50 μm.

Skeletal abnormalities are hallmark symptoms of progeria, which progress to skeletal dysplasia in progeria patients [33]. To evaluate the effects of *LMNA* deficiency on the bone, skeleton X-ray absorptiometry and histological assessments of *LMNA-KO* and WT rabbits were performed. Bone-length measurements confirmed that the average length of the femur (3.0±0.13) and tibia (3.5±0.14) in *LMNA*-KO rabbits was significantly shorter compared to that of the femur (4.1±0.12) and tibia (4.2±0.15) in WT rabbits (Fig. 5A-D). Thus, these results confirmed that *LMNA*-KO rabbits demonstrated severe growth retardation. Histological changes of the cortical bone, diaphysis, wrist and growth plate, are shown in Fig. 5E, F, compared with WT littermates, *LMNA*-KO rabbits exhibited decreased cortical bone width (Fig. 5Eb), significantly reduced numbers of osteoblasts and osteocytes (Fig. 5Ed), a rough articular surface (Fig. 5Fb) and irregular arrangement of the growth plate with increased porous areas (Fig. 5Fd). Combined, these results suggested that lamin A/C play important roles in bone metabolism.

**Figure 6.**
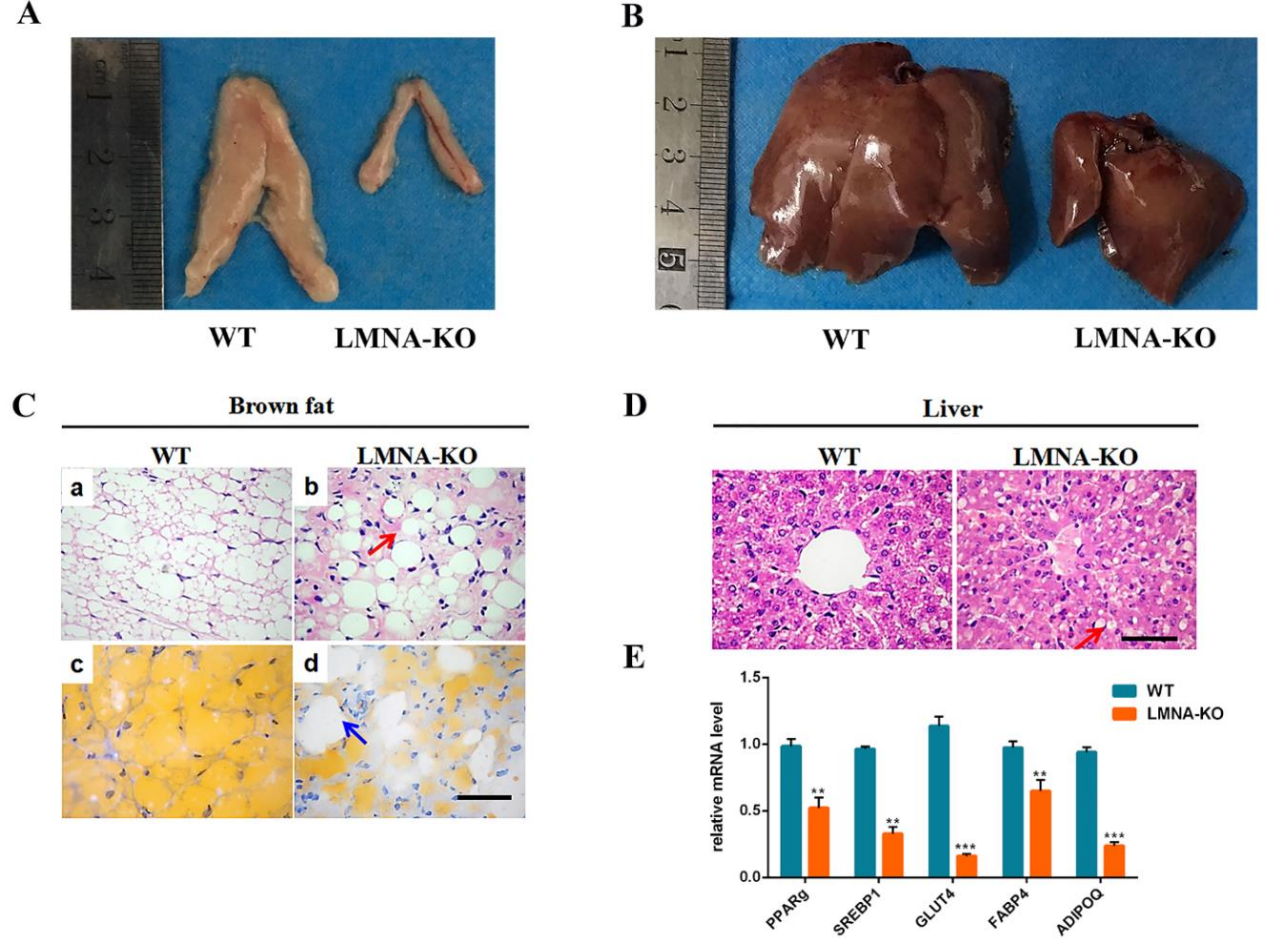
Lipodystrophy of *LMNA*-KO rabbits. (A) Dysplasia of the adipose tissue in *LMNA*-KO rabbits by anatomy analysis. (B) The liver of *LMNA*-KO rabbits and WT controls. (C) H&E-staining and Oil red staining of the brown adipose tissue from *LMNA*-KO rabbits and WT rabbits. (D) H&E-staining of liver sections from WT and *LMNA*-KO rabbits. (E) Gene expression of *PPARɡ*, *SREBF1*, *GLUT4*, *FABP4*, and *ADIPOQ* was determined by qRT-PCR. WT, WT control; *LMNA*-KO, *LMNA* gene knockout rabbit. All experiments were repeated for three times for each gene. Data are presented as the mean ± SEM and analyzed by t-tests using Graphpad Prism software 6.0. * *p* < 0.05; ** *p* < 0.01; *** *p* < 0.001. Scale bar, 50 μm.

### Lipodystrophy of LMNA-KO rabbits

In progeria patients, the loss of subcutaneous fat is significant [34]. Therefore, for the assessment of any adipose tissue abnormalities, we performed gross status examination as well as serum biochemical and histological analysis of adipose and liver tissue. As shown in Fig. 6A, B, a significantly reduction in fat tissue was observed in *LMNA*-KO rabbits when compared with WT littermates, but, liver was no significant differences between WT and *LMNA*-KO rabbits. Moreover, to investigate whether fat loss and hepatic steatosis were a primary effect on the lipid metabolism, the serum total cholesterol, high-density lipoprotein (HDL), low-density lipoprotein (LDL) cholesterol and triglyceride levels were determined. The data obtained from serum biochemical analysis indicated that increased levels of total cholesterol, HDL and LDL cholesterol, while decreased levels of triglyceride in *LMNA*-KO rabbits when compared with WT rabbits (Table 3). In addition, histological analysis showed increase in interstitial cells (Fig. 6Cb), vacuoles in fat cells (Fig. 6Cd), and fat infiltration in the liver (Fig. 6D) in *LMNA*-KO rabbits. Hence, our results suggested that defects of the *LMNA* gene caused lipodystrophy in *LMNA*-KO rabbits.

Mutations in the *LMNA* gene resulted in lipodystrophy, which directly affected lipid metabolism and storage in clinical [35]. To study abnormal lipid metabolism and storage, lipodystrophy related genes including SRE*BP1*, *PPARɡ*, *GLUT4*, *FABP4* and *ADIPOQ* were compared between *LMNA*-KO and WT rabbits. As shown in Fig. 6E, the expression of *PPARɡ*, *GLUT4*, *FABP4, SREBP1* and *ADIPOQ* genes was was significantly decreased in *LMNA*-KO rabbits when compared to WT controls, indicating that the *LMNA* may play a role in lipid metabolism and storage.

**Table 3:** Serum biochemical analysis in WT and *LMNA*-KO rabbits.

Biochemical indexes	8-Day		16-Day	
	WT controls	LMNA-KO	WT controls	LMNA-KO
T. Chol (mmol/l)	1.90 ± 0.37	7.77 ± 0.39^a^	2.72 ± 0.25	9.86± 1.73^a^
Triglycerides (mmol/L)	2.37 ± 0.20	1.35 ± 0.12^a^	1.97 ± 0.23	0.82±0.25^a^
HDL (mmol/L)	0.65± 0.13	1.37 ± 0.05^a^	0.71 ± 0.07	2.24± 0.17^a^
LDL (mmol/L)	1.26 ± 0.31	4.66 ± 0.56^a^	1.37 ± 0.15	4.94 ± 0.44^a^

a Different superscripts indicated significant difference (p < 0.05). Data are presented as mean ± SEM and analyzed by t-tests using Graphpad Prism software 7.0. * p < 0.05; ** p < 0.01; *** p < 0.001.

### Histological changes in lung and kidney of LMNA-KO rabbits

To examine whether *LMNA* deficiency had an impact on lung and kidney, histological and serum biochemical analysis were performed between *LMNA*-KO and WT rabbits. As shown in Supplementary Fig. 4A and B, no significant differences were observed between lung and kidneys from WT and *LMNA*-KO rabbits. However, *LMNA*-KO rabbits demonstrated that lymphocyte infiltration and alveolar septum thickening of lung (Supplementary Fig. 4C), renal tubular epithelial cell vacuolization and nuclear pyknosis in the kidney (Supplementary Fig. 4D).

To ascertain whether *LMNA*-KO rabbits exhibited clinical and serological features of some laminopathies, the serum biochemical analyses were compared between WT and *LMNA*-KO rabbits. As shown in Supplementary Fig. 4E-H, *LMNA*-KO rabbits exhibited significantly elevated serum cystatin c and alkaline phosphatase levels, while levels of serum albumin, total protein and creatinine were reduced (*p* < 0.05). These results confirmed an abnormal histology of lung and kidney tissues, including changes of clinical serum levels in the *LMNA* deficient rabbits.

### Cellular vacuolization and atrophy of LMNA-KO rabbits

To study whether mutations in the *LMNA* gene affected other organs, histological analysis of the stomach, eye, brain, and ear were performed and compared between *LMNA*-KO and WT rabbits. The results showed a typical phenotype of epithelial cell vacuolization in the stomach, lens, cornea and retina in *LMNA*-KO rabbits (Supplementary Fig. 5A). In addition, compared with WT littermates, *LMNA*-KO rabbits demonstrated cellular atrophy in cortical, hippocampal, and ear tissues (Supplementary Fig. 5B). These results suggested that *LMNA* deficiency caused changes of cellular vacuolization and atrophy in the stomach, eye, brain, and ear.

## DISCUSSION

In the present study, we developed a novel *LMNA* knockout rabbit models via zygote injection of Cas9 mRNA and a pair of sgRNA targeting exon 3 of the *LMNA* gene. We demonstrated that *LMNA*-KO rabbits exhibited almost all the hallmarks of the disease observed in the premature aging syndrome patients, including muscular dystrophy, cardiomyopathy, bone deficiency, lipodystrophy, and progeria. Moreover, in *LMNA*-KO rabbits, pathological changes of the liver, lung, kidney, stomach, lens, cornea, and retina tissues were observed. To our knowledge, this is the first report of an *LMNA* deficient rabbit model that resembles the human premature aging syndrome from gene mutation to clinical features.

To simulate human pathological conditions, we specifically generated an *LMNA* mutation in the rabbit genome. In this study, about 94% of the live pups carried mutations in target sites of the *LMNA* gene and about 91% of these targeted rabbits carried biallelic mutations. Mutations with fragment deletions or insertions, disrupted the reading frame of the *LMNA* gene, which was consistent with the findings presented in a previous study in which was reported that cytoplasmic injection of sgRNA-directed CRISPR/Cas9 mRNA could be used as an efficient approach to generate targeted knockout rabbits [36].

In this study, *LMNA* deficient rabbits, although not carrying human specific *LMNA* mutations, such as G608G (GGC>GGT), E145K (GAG>AAG), and G608S (GGC>AGC), develop several features of human premature aging syndrome and may therefore represent a promising model of premature aging syndrome [11, 33, 37, 38]. Regarding striated muscle laminopathy, the histological changes in cardiac muscles, skeletal muscles, fat, and bone also observed in *LMNA*-KO rabbits. These rabbits displayed typical phenotypes of laminopathies, including cardiomyopathy, muscular dystrophy, lipodystrophy, and osteodysplasia, and were consistent with the results described in other mouse models [21]. Cardiac dysfunction and interstitial fibrosis in patients have been reported by cardiac pathological analysis [17, 39]. Similarly, changes of cardiac pathology were observed in our *LMNA*-KO rabbits as well as in Lmna^-/-^ mice [40], suggesting that *LMNA*-KO rabbits mimic cardiac related disease of patients. Moreover, significant features of dystrophic and fibrosis were observed in gastrocnemius muscles, and diaphragm muscles of *LMNA*-KO rabbits, which have not been reported in patients, possibly due to the lack of such analysis in live individuals.

Additionally, different from the mouse model, *LMNA*-KO rabbits showed biochemical features of lipodystrophy including loss of subcutaneous fat. This is a typical characteristic of lipodystrophy but has not been reported in *LMNA*^L530P/L530P^ mice. In addition, serum levels of total cholesterol, HDL, and LDL cholesterol as well as triglyceride levels were altered in *LMNA*-KO rabbits, which have been reported in patients with familial partial lipodystrophy [41]. Interestingly, serum levels of cystatin c and albumin levels, important biological evidence of hepatic and renal dysfunction, were also changed in *LMNA*-KO rabbits, which were reported in patients [42, 43], while not determined in the mouse model. Thus, results obtained by *LMNA*-KO rabbits were represented human disease but were not observed in *LMNA*-KO mice. This may, possibly be because of the similarity in physiology between rabbits and humans. In addition, previous study has been showed that alterations of lamin A/C binding to *SREBP1* may be proposed as a possible factor in the development of familial lipodystrophy [44]. In the present study, we found that *LMNA*-KO rabbits had decreased *SREBP1* levels in brown fat, indicating that a possible mechanism of lipodystophy is due to the *SREBP1* dysfunction.

Actually, progeria is a rare deadly syndrome, it is characterized by severe growth retardation, loss of subcutaneous fat, bone and joint abnormalities, muscular dystrophy and cardiovascular pathology in mouse models and human clinical [19, 34, 45]. Consistent with the results described in both progeria patients and mice with mutations in the *LMNA* gene [20], *LMNA*-KO rabbits were indistinguishable from WT littermates at birth. However, 6-8 days after birth, *LMNA*-KO rabbits developed severe growth retardation, and died within 22 days. *LMNA*-KO rabbits died a few days earlier when compared to *LMNA*^L530P/L530P^ mice (4-6 weeks). In addition, cardiomyopathy was found in all death *LMNA*-KO rabbits. Therefore, we speculated that a possible cause of death involved cardiomyopathy, which has frequently been reported in mouse models and clinical [21, 46]. Furthermore, *LMNA*-KO rabbits demonstrated immobility of the joints, similar to ‘horse-riding stance’ symptoms present in the progeria patients [29]. The decreased number of eccrine grand is a sign of premature ageing and occurs in the skin, which has been reported in Lmna^L530P/L530P^ mice and in clinical [20], also found in this *LMNA*-KO rabbit.

In addition to skeletal muscles, the cardiac muscle, fat and bone defects, *LMNA*-KO rabbits exhibited detectable pathological changes in the liver, kidney, lung, stomach, lens, cornea and retina, which are proficient in the progeria patient [38, 47]. Specifically, *LMNA*-KO rabbits exhibited fat infiltration in the liver, pneumonia symptoms, alveolar septum thickening, and renal tubular epithelial cell vacuolization with nuclear pyknosis. Furthermore, typical epithelial cell vacuolization was observed in the stomach, lens, cornea, and retina of *LMNA*-KO rabbits. In addition, *LMNA*-KO rabbits showed signs of cellular atrophy, which was observed in cortical, hippocampal, and ear tissues. One possibility may be that the *LMNA* gene is a sign for terminal differentiation, given that many cells express *LMNA* during development [48]. Therefore, our novel results in *LMNA*-KO rabbits suggested that premature aging syndrome is a complex disorder with combined pathogenesis affecting different tissues in the body.

In the life cycle of an organism, aging and death are unavoidable events that occur within a few decades. Ageing, the progressive and irreversible loss of physiological integrity, is an extremely complex process that begins with fertilization and ends with death involving environmental and genetic factors [49]. The complex process of aging initially involves early aging including DNA damage or altered DNA that responds as mediators of aging and age-associated diseases [50]. Next, persistent growth failure occurred with poor postnatal growth that ends with early death from complications of atherosclerosis, such as myocardial infarction, stroke, atherosclerosis, or heart failure. Therefore, a progeria rabbit model is a valuable model for study aging-related syndromes and for elucidating the mechanisms underlying the normal process of aging in humans.

Taken together, to the best of our knowledge, this is the first report of a rabbit *LMNA*-KO model that shows close resemblance to a human disease. This novel rabbit model may facilitate understanding the process of premature aging syndrome and be beneficial for the development of novel therapeutic strategies to treat this devastating disease.

## Supplementary Materials

The Supplemenantry data can be found online at: www.aginganddisease.org/EN/10.14336/AD.2018.0209


