# Supplementary material for: LMNA-mutated Rabbits: A Model of Premature Aging Syndrome with Muscular Dystrophy and Dilated Cardiomyopathy
Source: Aging Dis. 2019 Feb 1;10(1):102–15. doi: 10.14336/AD.2018.0209 (PMC6345340; doi:10.14336/AD.2018.0209)
Supplement: Supplementary file 1 [file AD-10-01-102-s.pdf]

SUPPLEMENTARY DATA

LMNA-mutated Rabbits: A Model of Premature Aging Syndrome with Muscular Dystrophy and Dilated Cardiomyopathy

Tingting Sui[1,#], Di Liu[1,#], Tingjun Liu[1], Jichao Deng[1], Mao Chen[1], Yuanyuan Xu[1], Yuning Song[1], Hongsheng Ouyang[1], Liangxue Lai[1,2,*], Zhanjun Li[1,*]

[1]Jilin Provincial Key Laboratory of Animal Embryo Engineering, Jilin University, Changchun 130062, China
[2]Key Laboratory of Regenerative Biology, and Guangdong Provincial Key Laboratory of Stem Cells and Regenerative Medicine, South China Institute for Stem Cell Biology and Regenerative Medicine, Guangzhou Institutes of Biomedicine and Health, Chinese Academy of Sciences, Guangzhou, Guangdong 510530, China
[#]These authors contributed equally to this work

© 2018. Sui T et al. Published online at http://www.aginganddisease.org/EN/10.14336/AD.2018.0209

SUPPLEMENTARY DATA

```
   CCAAGAAGGAGGGTGACTTGATGGCCGCCCAGGCCCGG//ACCGCTCTCAGTGAGAAGCGCACGCTGGAGGGCGA WT
16 CCAAGAAGGAGGGTGACTTGATGGCCG-----------//--------------------CACGCTGGAGGGCGA -79

17 CCAAGAAGGAGGGTGACTTGATGGC-------------//----------------------CGCTGGAGGGCGA -83

18 CCAAGAAGGAGGGTGACTTGATGGC-------------//----------------------CGCTGGAGGGCGA -83
   CCAAGAAGGAGGGTGACTTGATGGCCG-----------//----------------------CGCTGGAGGGCGA -81

19 CCAAGAAGGAGGGTGACTTGATGGCCG-----------//----------------------CGCTGGAGGGCGA -81
   CCAAGAAGGAGGGTGACTTGATG---GCCC--------//------------------------CTGGAGGGCGA -83

20 CCAAGAAGGAGGGTGACTTGATGGCCG-----------//----------------------CGCTGGAGGGCGA -81
   CCAAGAAGGAGGGTGACTTGATG---------------//-----------------------GCTGGAGGGCGA -86
   CAAGAAGGAGGGTGACTTGATGG-----------CCGG//ACCGCTCTCAGTGAGA-------GCTGGAGGGCGA -17

21 CCAAGAAGGAGGGTGACTTGATGGC---------CCGG//ACCGCTCTCAG-----------CGCTGGAGGGCGA -20
   CCAAGAAGGAGGGTGACTTGATGGCCG-----------//----------------------CGCTGGAGGGCGA -81

22 CCAAGAAGGAGGGTGACTTGATGGC-------------//----------------------CGCTGGAGGGCGA -83
   CCAAGAAGGAGGGTGACTTGATGGCCGCCCAGGCCCGG//ACCGCTCTCAGTGAGAAGCGC----TGGAGGGCGA -4

23 CCAAGAAGGAGGGTGACTTGATGG----------CCGG//----------------------CGCTGGAGGGCGA -40
   CCAAGAAGGAGGGTGACTTGATGGCCGC----------//----------------------CGCTGGAGGGCGA -80
24 CCAAGAAGGAGGGTGACTTGATGGCCGCCCAGGCCCGG//ACCGCTCTCAGTGAGAAGCGCACGCTGGAGGGCGA WT
   CCAAGAAGGAGGGTGACTTGATGGCCG-----------//--------------GAAGCGCACGCTGGAGGGCGA -73

25 CCAAGAAGGAGGGTGACTTGATGGCC----------GG//ACCGCTCTCAGTGAGAA------GCTGGAGGGCGA -16
   CCAAGAAGGAGGGTGACTTGATGGCCG-----------//---------------------ACGCTGGAGGGCGA -81

26 CCAAGAAGGAGGGTGACTTGAT--------------GG//ACCGCTCTCAGTGAGAAGCGC----TGGAGGGCGA -18
   CCAAGAAGGAGGGTGACTTGATGGCCG-----------//----------------------CGCTGGAGGGCGA -81

27 CCAAGAAGGAGGGTGACTTGATGGCC---------CGG//ACCGCTCTCAGTGAGAAGCGC----TGGAGGGCGA -13
   CCAAGAAGGAGGGTGACTTGATGGCT------------//----------------------CGCTGGAGGGCGA -80

28 CCAAGAAGGAGGGTGACTTGATGGCCGC----------//----------------------------------- -127
   CCAAGAAGGAGGGTGACTTGATGGCCG--------CGG//ACCGCTCTCAGTGAGAA------GCTGGAGGGCGA -15

29 CCAAGAAGGAGGGTGACTTGA-----------------//-------------------------TGGAGGGCGA -90

30 CAAGAAGGAGGGTGACTTGATGGCCG------------//----------------------CGCTGGAGGGCGA -81
   CCAAGAAGGAGGGTGACTTGATGGCC------------//--------------------------GGAGGGCGA -86

31 CCAAGAAGGAGGGTGACTTGATGGCC---------CGG//ACCGCTCTCAGTGAGAAGCGC----TGGAGGGCGA -13
   CCAAGAAGGAGGGTGACTTGATGGCCG-----------//------------GAGAAGCGCACGCTGGAGGGCGA -71
   CCAAGAAGGAGGGTGACTTGATGGC-------------//-------------------GCACGCTGGAGGGCGA -83

32 CCAAGAAGGAGGGTGACTTGATGGCCGCCCAGGCCCGG//ACCGCTCTCAGTGAGAAGCGCACGCTGGAGGGCGA WT
```

S

upplementary Figure 1. Mutation detection of the 16-32 F0 rabbits by T-cloning and Sanger sequencing. The sgRNA sequences are shown in red; PAM sites are underlined and highlighted in green; insertions are shown in blue; deletions (-); WT, wild-type control.

© 2018. Sui T et al. Published online at http://www.aginganddisease.org/EN/10.14336/AD.2018.0209

SUPPLEMENTARY DATA

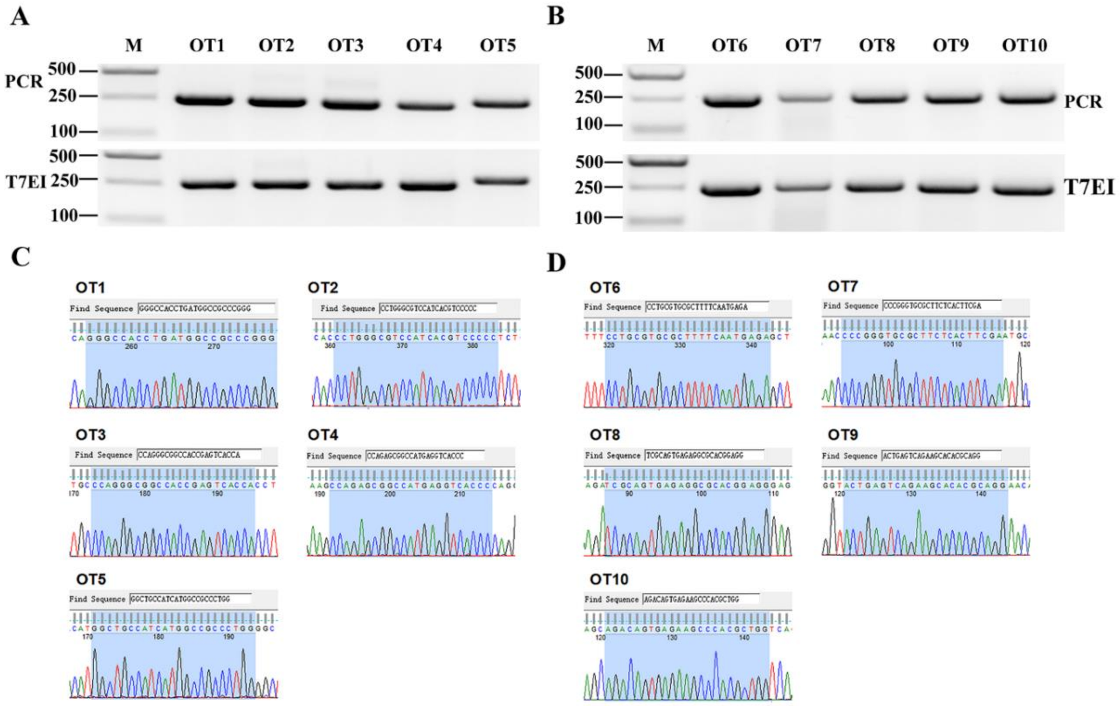

Supplementary Figure 2. Off-target analysis of *LMNA*-KO rabbit. The T7E1 cleavage analysis of five potential off-target sites (POTS) for sgRNA1 (A) and sgRNA2 (B). M, DL2000; OT1-OT5 represented the five POTS for sgRNA1 and OT6-OT10 represented the five POTS for sgRNA2. T-cloning and Sanger sequencing of the five POTS for sgRNA1 (C) and the five POTS for sgRNA2 (D), the region included the 20bp of the POTS and PAM were shown in blue shadow.

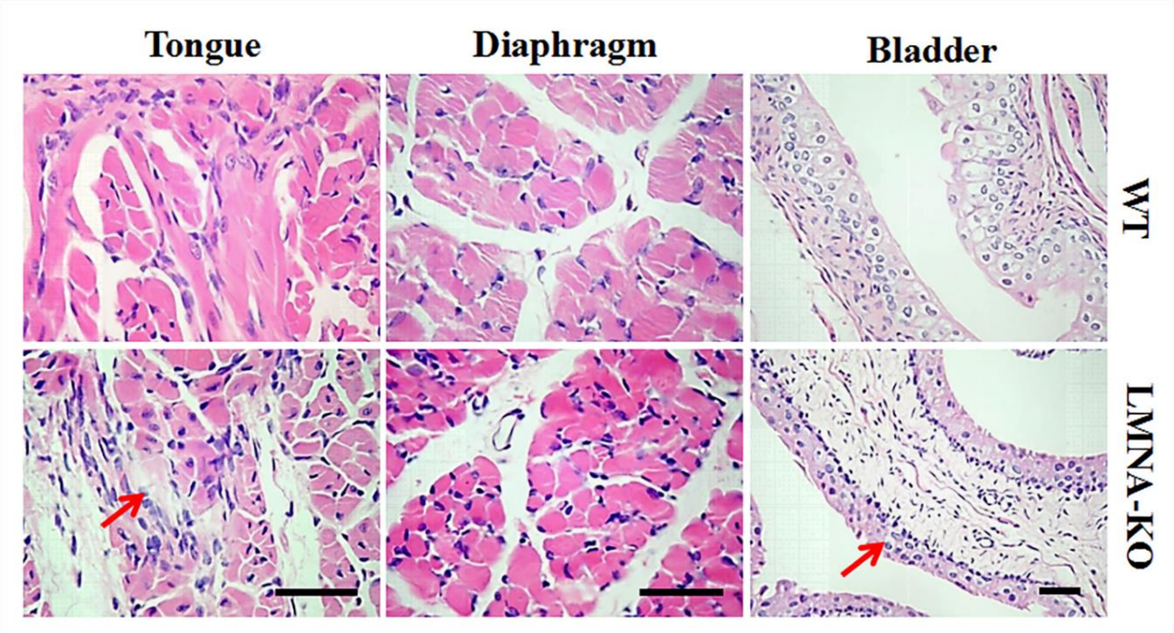

Supplementary Figure 3. H&E-staining of the tongue, diaphragm and bladder. H&E-staining of tongue, diaphragm, and bladder muscles from WT and *LMNA*-KO rabbits. Scale bar, 50 µm.

© 2018. Sui T et al. Published online at http://www.aginganddisease.org/EN/10.14336/AD.2018.0209

SUPPLEMENTARY DATA

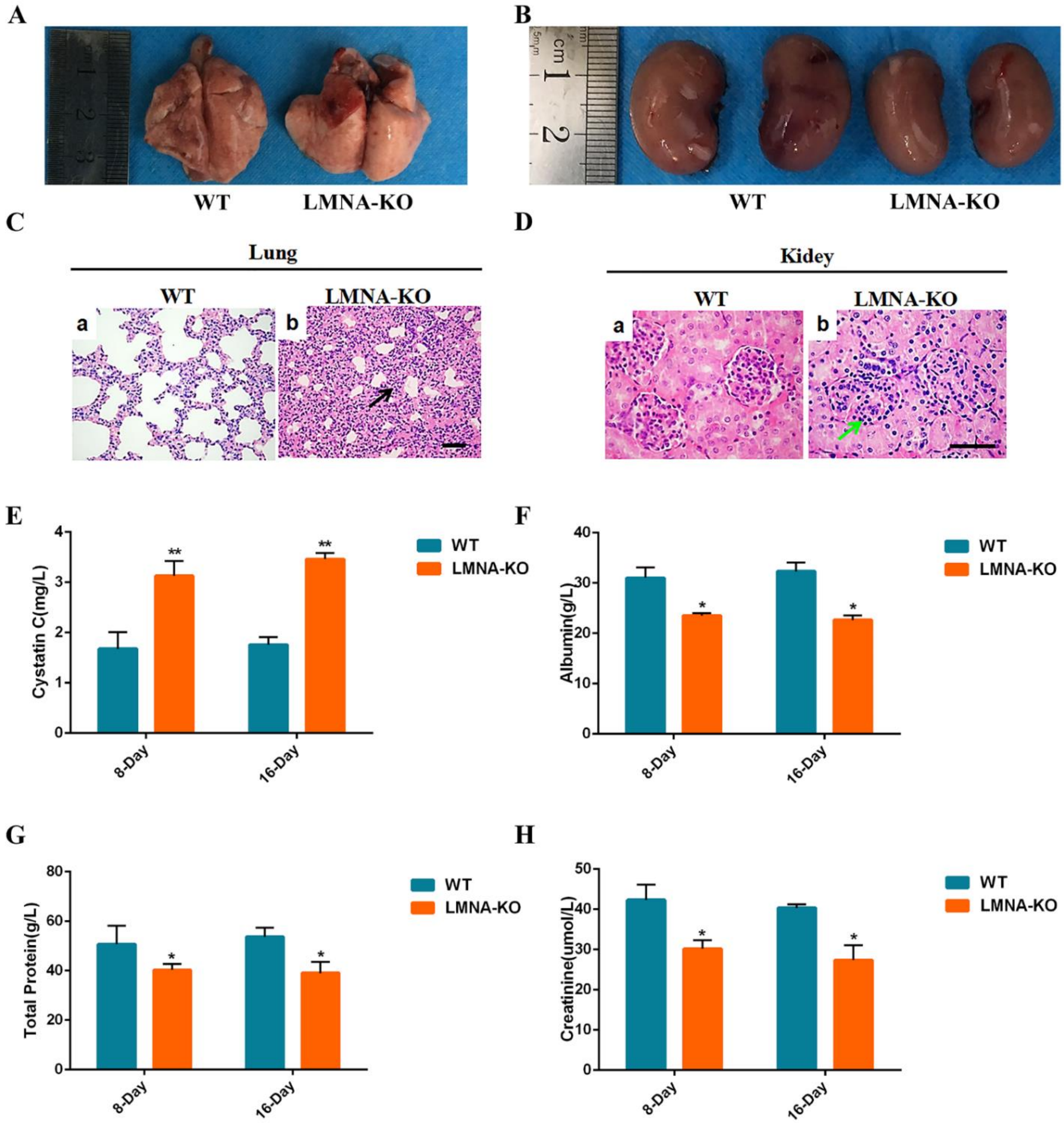

Supplementary Figure 4. Histological changes of lung and kidney in *LMNA*-KO rabbits. The lung (A) and kidney (B) of *LMNA*-KO rabbits and WT controls. (C) H&E-staining of lung from WT and *LMNA*-KO rabbits. (D) H&E-staining of kidney sections derived from WT and *LMNA*-KO rabbits. Significantly increased cycstatin C levels (E), significantly decreased albumin levels (F), total protein levels (G) and creatinine levels (H) in *LMNA*-KO rabbits compared with WT rabbits. Scale bar, 50 μm.

Supplementary Movie 1. The movie of WT rabbit.

Supplementary Movie 2. The movie of *LMNA*-KO1 rabbit.

Supplementary Movie 3. The movie of *LMNA*-KO2 rabbit.

© 2018. Sui T et al. Published online at http://www.aginganddisease.org/EN/10.14336/AD.2018.0209

SUPPLEMENTARY DATA

Supplementary Table 1. ***The primers of potential off-target sites (POTS) used in this study.***

	Potential Off Target Site	Number of mismatch	Position	PCR Primer
s1	GGGCCACCTGATGGCCGCCCGGG	3	chrUN0:+348346	F：CCGGCCAGGTGAGAAGAAA<br>R：TAACAGTGTCTGAGGCGTGTG
	GGGGGACGTGATGGACGCCCAGG	3	chr9:-110600689	F：GCTGTCATATGCTACCCTTCAG<br>R：GACCATCACCTCCACAAGAAG
	TGGTGACTCGGTGGCCGCCCTGG	3	chrUN0:-97332	F：CTGGGCAGATCAAGACTGAA<br>R：GCCTCTGCACATACTGACTAA
	GGGTGACCTCATGGCCGCTCTGG	3	chrUN0:-12139	F：GTGCAGTGGATGTGAGTTTG<br>R：CAGCAAACGTGCCTGAAAT
	GGCTGCCATCATGGCCGCCCTGG	4	chrUN0:+1372005	F：TACGGTCTCCTTCAAGGTCT<br>R：TCCGGAAGATACTCCCAAAGA
**s2**	TCTCGTTGAAAAGCGCATGCAGG	4	chr2:-38825143	F：GACACACAGTTGCAGAGTTAG<br>R：GGAACAGGGCTAGAGAGAAT
	TCGAAGTGAGAAGCGCACCCGGG	3	chrUN0:-53508	F：CTACAGTGGACACACATCAC<br>R：CTTGCTTGAAGACGGAGTATAA
	TCGCAGTGAGAGGCGCACGGAGG	3	chr9:+87371194	F：TGACATGCTGTTGGAGACTA<br>R：CTTCCCGAGTATTTGCTGTTAC
	ACTGAGTCAGAAGCACACGCAGG	4	chrUN0:+660880	F：GCAGATCATGAAGGCAGAAG<br>R：ATTCTCATCCCTTTCCCTAAGA
	AGACAGTGAGAAGCCCACGCTGG	4	chr21:+7109152	F：TCAGGAGTCATGGAGTGATTT<br>R：TGGAGTCGCTAAGAATGGG

The mismatches of sgRNA were shown in red. s1, sgRNA1; s2, sgRNA2.

Supplementary Table 2. Primers for qPCR analysis

Name	Primers	Sequence (5’-3’)	Produce size (bp)
FABP4	FABP4-F	ACCATTAAATCAGAGAGCACCTT	99
	FABP4-R	GCTCTTGACTTTCCTGTCATCT	
ADIPOQ	ADIPOQ-F	CTTGTTGGTCCTAAGGGTGAC	102
	ADIPOQ-R	TTCTCCGGGCTCTCCTTT	
SREBP1	SREBP1-F	ACAGACAAACTGCCCATCC	113
	SREBP1-R	GAACGGTAGCGCTTCTCAAT	
GLUT4	GLUT4-F	GTTCCTCATTGGCGCCTACTC	116
	GLUT4-R	CGATGGCCAGCTGGTTGAG	
PPARg	PPARg-F	GCATCTCTGCTCCACACTATG	115
	PPARg-R	GGTTCCACTTTGATTGCACTTT	
GAPDH	GAPDH-F	ATCCATTCATTGACCTCCACTAC	116
	GAPDH-R	GTACTGGGCACCAGCATCAC	

© 2018. Sui T et al. Published online at http://www.aginganddisease.org/EN/10.14336/AD.2018.0209
